# Pure Red Cell Aplasia with Del(20q) Sensitive for Immunosuppressive Treatment

**DOI:** 10.1155/2020/1262038

**Published:** 2020-01-20

**Authors:** Anh Khoi Vo, Hilde Kollsete Gjelberg, Randi Hovland, Marte Karen Lindstad Brattås, Øystein Bruserud, Håkon Reikvam

**Affiliations:** ^1^Section Hematology, Department of Medicine, Haukeland University Hospital, Bergen, Norway; ^2^Department of Pathology, Haukeland University Hospital, Bergen, Norway; ^3^Department of Medical Genetics, Haukeland University Hospital, Bergen, Norway; ^4^Department of Biological Sciences, University of Bergen, Bergen, Norway; ^5^Department of Medicine, Haraldsplass Deaconess Hospital, Bergen, Norway; ^6^Department of Clinical Science, University of Bergen, Bergen, Norway

## Abstract

Pure red cell aplasia (PRCA) is a rare syndrome that only affects the erythroid lineage. It is defined by a normocytic, normochromic anemia with a marked reticulocytopenia and severe reduction or absence of erythroid precursors in the bone marrow. Treatment of primary, idiopathic PRCA is immunosuppressive therapy. Although it is rare, isolated cytogenetic abnormalities can be seen in PRCA, and abnormal karyotype is associated with poor response to immunosuppressive therapy and poor prognosis. We describe a 77-year-old male with primary, idiopathic PRCA and a deletion of chromosome 20q, del(20q), in the bone marrow cells. He was successfully treated with immunosuppressive therapy and became transfusion-independent. The same cytogenetic abnormality has also been described in a few other reports; taken together, these observations suggest that del(20q) may represent a recurrent cytogenetic abnormality in PRCA. Our case report clearly illustrates that even patients with primary PRCA and an abnormal karyotype can respond to immunosuppression and become transfusion-independent.

## 1. Introduction

Pure red cell aplasia (PRCA) is a rare syndrome that solely affects erythroid lineage. It is defined by a normocytic, normochromic anemia with a marked reticulocytopenia and severe reduction or absence of erythroid precursors from the bone marrow [1, 2]. The disease is classified into congenital (also known as Diamond-Blackfan anemia) and acquired PRCA. The acquired form is usually an acute and self-limiting disease that mainly occurs in children, whereas the chronic variant is most common in adults. Although acquired PRCA in adults may present as a primary, idiopathic disease, it can also be secondary to other underlying conditions.

The primary form of PRCA is considered to be an autoimmune disease with immune-mediated inhibition of the differentiation and maturation of erythroid precursors [1–3]. On the contrary, secondary PRCA can be associated with various disorders including lymphoproliferative disorders (e.g., leukemia, Hodgkin's and non-Hodgkin's lymphoma, and thymoma), solid tumors, viral infections (e.g., parvovirus B19 infections), other autoimmune disorders, and certain pharmacologic agents [1, 2]. Although usually not considered to be a preleukemic condition [2], it may be a prodrome to myelodysplastic syndrome (MDS) [4, 5]. Several case reports have described a number of recurring cytogenetic aberrations, e.g., isolated i(17q) and del(5q); most of these cases are patients with MDS with PRCA(5). Isolated del(20q) has also been reported in cases of both PRCA with MDS and primary, idiopathic PRCA [4, 6]. Taken together, these previous reports indicate a potential association between PRCA and certain cytogenetic abnormalities. Here, we describe a case of PRCA with an isolated del(20q) with no evidence for any concomitant hematologic disorders.

## 2. Case Presentation

A 77-year-old man was undergoing follow-up at his primary hospital due to chronic kidney disease stage 4. In addition, he had abnormal levels of liver and pancreas serum markers of unknown etiology. His medical history included hypertension, hypercholesterolemia, Barrett's esophagus, and stenting of the left carotid artery due to a transient ischemic attack. During routine follow-up, blood tests revealed a progressive normocytic, normochromic anemia. The patient did not respond to the initial treatment with iron supplements and erythropoietin injections. There was a gradual progression until the blood tests showed hemoglobin (Hb) 6.0 g/dL (normal range: 13.4–17.0), mean corpuscular volume (MCV) 101 fL (82–98), reticulocytes <0.010 × 10^12^/L (0.03–0.1), thrombocytes 445 × 10^9^/L (145–348), and total leukocytes 6.8 × 10^9^/L (3.5–11.0). The peripheral blood differential count showed neutrophils, 4.8 × 10^9^/L (1.7–8.2), lymphocytes, 0.9 × 10^9^/L (0.7–5.3), monocytes, 0.7 × 10^9^/L (0.04–1.30), eosinophils, 0.4 × 10^9^/L (0.0–0.7), and basophils, <0.1 × 10^9^/L (0.0–0.3). Thus, the patient had a normocytic, normochromic anemia with low reticulocyte counts but no evidence for a general bone marrow failure.

**Figure 1:**
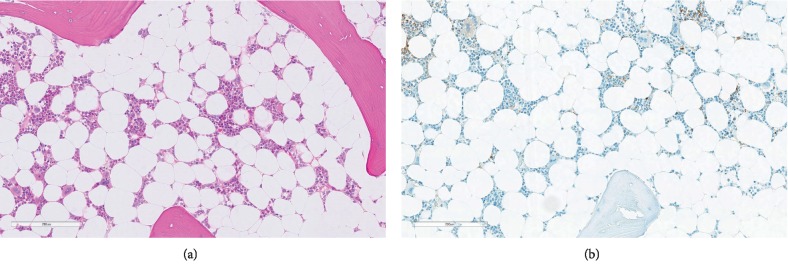
Histopathological features of the bone marrow in PRCA. (a) The bone marrow core biopsy section shows a slightly hypocellular marrow with intact granulocytic and megakaryocytic cells but the absence of erythroid colonies (hematoxylin and eosin, scale bar: 200 *μ*m). (b) Immunoperoxidase staining for hemoglobin A highlights only scattered positive cells (dark brown) with no colony formation (scale bar: 200 *μ*m). Most of the brownish appearing cells represent iron-laden macrophages.

**Figure 2:**
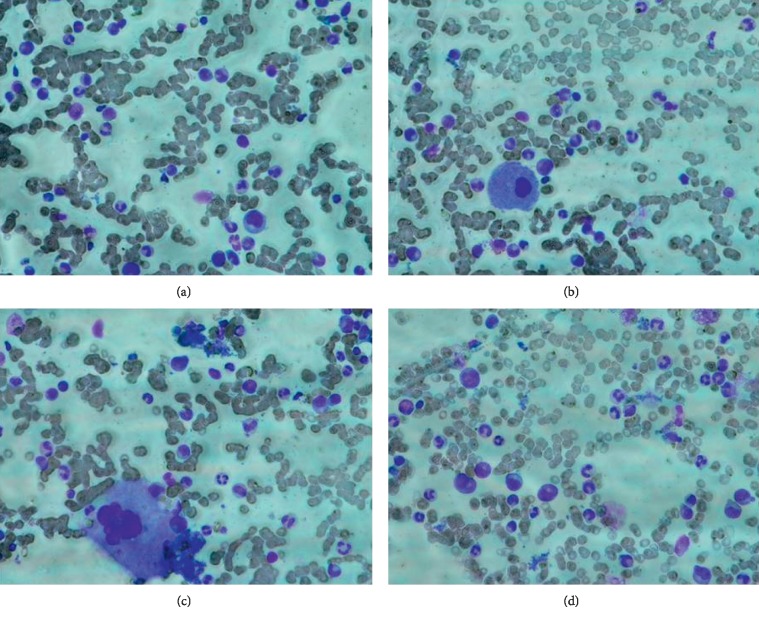
Cytomorphology of the bone marrow aspirate. Bone marrow aspirate at the time of diagnosis was stained with May–Grunwald–Giemsa (MGG). The smear confirmed the total absence of erythropoiesis, without signs of dysplasia in granulocytopoiesis or megakaryocytopoiesis. Four different areas are demonstrated in the figure.

A bone marrow biopsy showed total absence of erythropoiesis with normal megakaryocytes and normal granulocytopoiesis with large amounts of iron in the bone marrow (Figure 1). This was also confirmed by cytomorphology of the bone marrow aspirate, demonstrating total absence of erythropoiesis, without signs of dysplasia in the granulocytopoiesis or megakaryocytopoiesis (Figure 2). No definitive signs of dysplasia were detected. Thus, absence of erythropoiesis was the only abnormality demonstrated by the bone marrow examination, and the patient was treated with regular erythrocyte transfusions.

**Figure 3:**
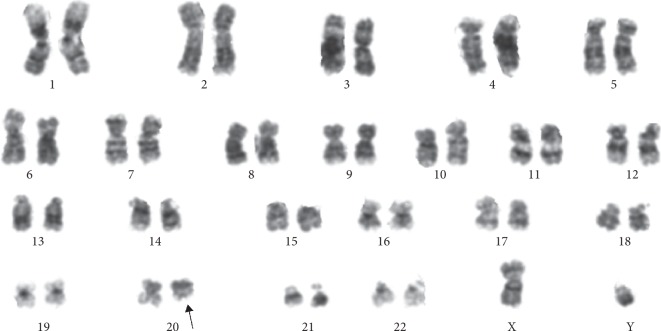
Karyotyping of bone marrow cells using G-banding. G-banding analysis after unstimulated culture of bone marrow showed the karyotype 46,XY del(20)(q11.2).

Further investigations included serological tests for B19 parvovirus, Epstein–Barr virus (EBV), cytomegalovirus (CMV), hepatitis A, hepatitis B, hepatitis C, and human immunodeficiency virus (HIV), but there was no evidence for any of these infections being the cause of the hematological disease. A computed tomography (CT) scan of the chest showed no signs of thymoma, and flowcytometric analyses of the bone marrow did not detect any evidence for monoclonal B-cell or T-cell populations. Karyotyping of bone marrow cells revealed a deletion at the long arm of chromosome 20 and the karyotype 46,XY,del(20) (q11)[6]/46,XY[14] (Figure 3).

**Figure 4:**
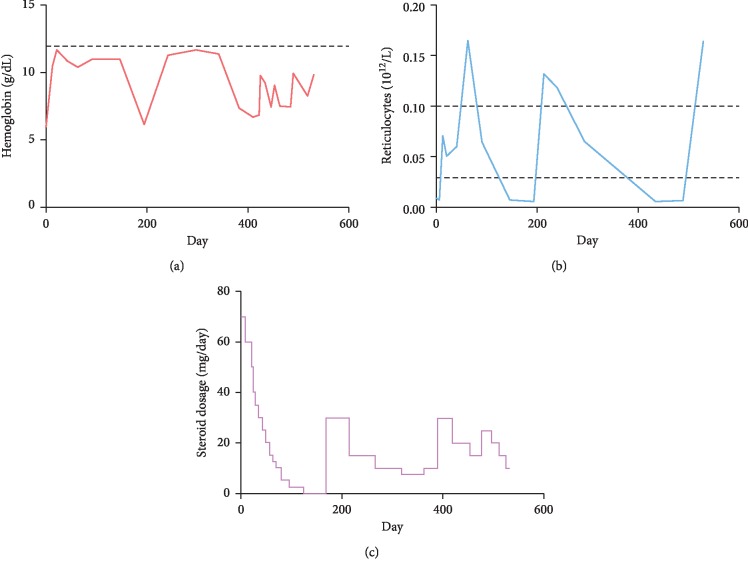
The response to immunosuppressive treatment. The figure shows the levels of hemoglobin (a) and reticulocytes (b) in peripheral blood after initiation of corticosteroid therapy. The corticosteroid treatment was gradually tapered (c). Days on the *X*-axis indicate days after initial diagnosis. The stippled horizontal lines indicate the normal range for hemoglobin and reticulocytes.

The findings justified the diagnosis of PRCA with the chromosomal abnormality del(20q). Previous studies suggest treatment with cyclosporine A to be superior to corticosteroids and cyclophosphamide with regard to response rate and relapse-free survival [1, 7]. This was not the preferred choice of treatment in the present case due to the patient's chronic kidney disease and the risk of nephrotoxicity. Oral prednisolone was therefore initiated with a dosage of 1 mg/kg/day in addition to regular erythrocyte transfusions (Figure 4). The patient responded to the steroid treatment; increasing peripheral blood reticulocyte counts were observed after two weeks, and after three weeks of treatment, the patient was transfusion-independent with stable hemoglobin levels corresponding to 10-11 g/dL. Initially, no major side effects of the steroid treatment were reported.

Tapering of corticosteroids was initiated after six weeks and was initially without any complications (Figure 4). Hemoglobin levels remained stable, although the reticulocyte counts decreased at a later point. After discontinuation of corticosteroids (Figure 4), the patient suffered a relapse and treatment was reinitiated but with a lower prednisolone starting a dose of 30 mg/day. He responded to this treatment, and the dose was slowly tapered over months until he could continue with a maintenance dose of prednisolone 7.5 mg daily. During this period, he had a stable hemoglobin level above 10 g/dL. However, the patient had a second relapse and was hospitalized for erythrocyte transfusions. A new bone marrow aspiration demonstrated normal granulocytopoiesis, normal megakaryocytes, and total absence of erythropoiesis, coinciding with the initial findings. Mutational analysis of the bone marrow with the Truesight Myeloid Sequencing Panel (Illumina®) did not reveal mutations and strengthened the suspicion of a nonclonal myeloid disorder. Hence, PRCA remained the most likely diagnosis. The patient had then used oral prednisolone for 16 months, and as the response to steroid treatment was assumed to decline and given the potential serious side effects of long term steroid treatment, we changed the immunosuppressive treatment to cyclosporine A with tapering of steroid treatment. He ultimately received cyclosporine A at a dose of 200 mg/day with careful monitoring of his kidney and liver functions. His reticulocyte count and hemoglobin level was rapidly increasing (Figure 4).

## 3. Discussion

In this case report, we present a PRCA patient with a deletion of chromosome 20q in the bone marrow cells, and no evidence was found for concomitant hematologic disorders or other disorders commonly associated with PRCA (e.g., viral infections and solid tumors) [1, 2]. The diagnosis was based on peripheral blood and bone marrow examination, immunohistochemistry, and G-banding analysis of bone marrow cells.

Although the associations with other conditions and differences in the responsiveness to various therapeutic strategies suggest a diverse pathophysiology of PRCA [1, 7, 8], the role of cytogenetic abnormalities in the pathogenesis of PRCA remains unknown. A small number of studies have reported PRCA patients with isolated cytogenetic abnormalities; most of these patients had PRCA together with other hematologic disorders. Inui et al. reported a case of isolated isochromosome i(17)q in MDS with PRCA; two similar cases had been reported earlier, and these findings suggest that i(17)q is a recurrent cytogenetic abnormality in MDS with PRCA. Another possible recurring abnormality described in MDS with PRCA is del(5q). Park et al. reported del(5q) in four of six patients with MDS with PRCA [9]. This abnormality is commonly seen in the context of MDS alone [5, 10, 11]. These MDS patients frequently show erythroid hypoplasia, i.e., a marked decrease and maturation arrest of erythroid precursors; such hypoplasia is uncommon in MDS and has been proposed to represent a distinct entity referred to as MDS with PRCA [4, 9, 12]. The present patient had no cytopenias in other lines, and he had no mutations in the most common genes associated with MDS. The diagnosis of MDS or development of frank MDS hence seems unlikely, strengthening the diagnosis of PRCA.

Our patient had an isolated deletion of chromosome 20q which has only been reported in a few cases of PRCA. Kurtin et al. reported two such PRCA cases with no other hematologic disorders, and they postulated an association between this karyotypic abnormality and PRCA [6]. Wang et al. [4] reported two PRCA patients with this abnormality together with MDS. Lacy et al. [11] also reported a PRCA patient with del(20q), but it is not clear whether this patient had any additional hematologic disorders.

The del(20q) abnormality is associated with dyserythropoiesis and dysmegakaryocytopoiesis and is thought to represent an early aberration in hematologic malignancies due to the loss of the *L3MBTL1* polycomb tumor suppressor protein; this has been shown to cause replicative stress and genomic instability *in vitro* [6, 10, 13]. Knockdown of *L3MBTL1* represses human-induced pluripotent stem cells (iPSCs) for hematopoietic differentiation and enhances commitment toward the erythroid lineage [14]. Del(20q) is more common in various myeloid disorders [6, 13, 15] and in the rare Shwachman–Diamond syndrome [16], whereas it is uncommon in aplastic anemia (AA) [17]. However, its role in the development of these disorders are yet to be further clarified. In a study with patients who acquired isolated del(20q) after cytotoxic therapy, approximately two-thirds did not develop therapy-related myeloid neoplasms. The subset of patients who developed therapy-related myeloid neoplasms often presented with del(20q) in a higher percentage of metaphases, terminal deletion rather than interstitial, and a longer persisting deletion [18]. The abnormality has been more extensively studied in MDS where it is associated with a favorable prognosis when no other cytogenetic abnormalities are present [19]. Besides *L3MBTL1*, it is not known which of the lost genes are involved in the development of MDS and other myeloid disorders [20]. At the time of diagnosis and after several relapses, our patient did not have any evidence for MDS or any other myeloid disorders, though it should be emphasized that some reports have suggested that PRCA might be a pre-MDS disorder [1, 4, 12, 21, 22].

PRCA is a very diverse and rare disease with no standardized therapeutic strategy. The treatment is mainly based on experience and case reports [8]. The first-choice treatment for primary, idiopathic PRCA is immunosuppressive therapy. Corticosteroids are usually preferred [1, 7, 23], and the reported response rates are 27–62%, but the relapse rate is substantial [1, 7, 11, 23]. Cyclosporine A has also been reported to be effective with a response rate of 65–87% [7], but the experience with this drug is more limited, and there are uncertainties regarding relapse rate. Maintenance therapy is required to prevent relapse for most patients. In spite of this, cyclosporine A is currently considered to be the first line of treatment granted among the patients who does not present with contraindications [7, 8, 24]. It has also been reported to be effective in patients with refractory PRCA [3]. In the present case, corticosteroid therapy was chosen due to chronic kidney disease of unknown etiology. Corticosteroid therapy also has a high rate of second remission in relapsed patients [25] and was successful in our patient even though it was reinitiated at a lower dose. However, immunosuppressive therapy generally seems to be less effective in relapsed PRCA compared with treatment naïve PRCA [26], and patients may develop refractory PRCA [3].

Monoclonal antibodies such as alemtuzumab and rituximab are other alternatives commonly used for patients with refractory PRCA. Varying results have been reported for these drugs [24, 27, 28], possibly due to the pathophysiological heterogeneity of PRCA with the majority of the idiopathic cases being T-cell-mediated, while cases responsive to rituximab may represent B-cell-mediated etiology [8].

PRCA with abnormal karyotype seems to be associated with poor response to immunosuppressive therapy and consequently poor prognosis. Lacy et al. [11] reported three patients with abnormalities involving chromosome 5 and one with del(20q), none of which responded to immunosuppressive treatment. Similar observations have been made by others [29, 30]. This lack of responsiveness possibly reflects the heterogeneity of PRCA patients with regard to the pathogenesis. In contrast to previous reports, our patient responded to two modalities of immunosuppression even after several relapses. However, there are few studies on idiopathic PRCA with isolated cytogenetic abnormalities, and our present case emphasizes the need for further studies to further elucidate the role of del(20q) in PRCA. Our case report clearly shows that response to immunosuppression is possible also for PRCA patients with cytogenetic abnormalities.

## 4. Conlusion

To conclude, PRCA is a rare disease, and cytogenetic abnormalities are detected for a subset of these patients. The role of such abnormalities in the pathogenesis of PRCA remains elusive. However, even patients with cytogenetic abnormalities may respond to immunosuppressive treatment and become transfusion-independent. For this reason, detection of an abnormal karyotype should not exclude immunosuppression as the first-line treatment.
